# An intelligent system for determining the degree of tree bark beetle damage based on the use of generative‐adversarial neural networks

**DOI:** 10.1002/pei3.70015

**Published:** 2024-11-01

**Authors:** Sineglazov Victor, Hordun Mykhailo, Junttila Samuli

**Affiliations:** ^1^ State University “Kyiv Aviation Institute” Kyiv Ukraine; ^2^ National Technical University of Ukraine “Igor Sikorsky Kyiv Polytechnic Institute” Kyiv Ukraine; ^3^ Glushkov Institute of Cybernetics of the National Academy of Sciences of Ukraine Kyiv Ukraine; ^4^ University of Eastern Finland School of Forest Sciences Joensuu Finland

**Keywords:** bark beetle, CNN, GAN, image classification, multispectral images, remote sensing

## Abstract

The increasing prevalence of tree bark beetle infestations poses a significant threat to forest ecosystems, leading to detrimental impacts on biodiversity, carbon storage, and timber resources. This study addresses the pressing need for innovative solutions to enhance forest management practices through early detection and assessment of tree health. We developed an intelligent system leveraging multispectral images and generative‐adversarial networks (GANs) to accurately determine the extent of bark beetle damage. Recognizing the challenges posed by traditional neural networks, which require vast amounts of labeled training data, we proposed a novel approach that utilizes a GAN architecture. In this system, a discriminator functions as a classifier, effectively trained on both real and synthetically generated data. Our methodology not only reduces the dependency on extensive labeled datasets but also enhances the robustness of the classification process. The results indicate a classification accuracy of 87.5%, demonstrating significant promise for improving detection capabilities even with limited training resources. The implications of this research are profound, offering potential benefits for the forestry industry through optimized management strategies and economic gains. Furthermore, our findings contribute to the preservation of critical ecosystem services by providing a means to monitor forest health effectively. However, the responsible implementation of this technology is paramount. Continuous refinement of the model, integration with traditional ecological knowledge, and ensuring transparency and equitable access to the developed system are essential for maximizing societal benefits. Additionally, addressing risks such as misinterpretation of data, overreliance on technology, and privacy concerns is crucial to minimize unintended consequences. Overall, this research presents a significant advancement in the field of forest health monitoring and establishes a foundation for future developments in intelligent ecological management systems.

## 1 INTRODUCTION

Bark beetles are a well‐known group of insects that colonize virtually all types of plant tissue and play important and diverse roles in ecosystem processes, contributing to biodiversity, nutrient cycling, and heterogeneity. However, some species of bark beetles (Coleoptera: Curculionidae, Scolytinae) can lead to the death of a significant number of trees in coniferous forests around the world (Vega & Hofstetter, 2014). In most cases, bark beetle outbreaks are natural disturbances in the ecology of coniferous forests, playing a crucial role in many ecological processes (Vega & Hofstetter, 2014). However, these outbreaks, which are among the most common natural disturbances in coniferous forests worldwide, often lead to large‐scale tree mortality at the landscape level (Kurz et al., 2008). This, in turn, can have significant economic and social consequences, affecting the forestry industry, increasing the risk of wildfires, and disrupting local ecosystems (Fettig et al., 2007; Hlásny et al., 2021). Furthermore, projections indicate that bark beetle disturbances in Europe are expected to increase sevenfold by 2030 compared to the period from 1971 to 1980 (Seidl et al., 2014). Therefore, accurate and timely detection of the initial stages of tree damage by bark beetles can be an important task to mitigate the consequences of damage, prevent the spread of bark beetles in large forest areas, and minimize economic losses (Fettig et al., 2007; Hlásny et al., 2021).

The damage to coniferous trees caused by bark beetles is divided into 4 phases based on changes in the crowns of affected trees: green, yellow, red, and gray phases. It is important to detect tree damage by bark beetles in the early (green) phase, which is a rather difficult task that requires field research. However, field research is expensive and time‐consuming when it comes to large areas of forests. Therefore, the use of Remote Sensing (RS) data together with Machine Learning (ML) or Deep Learning (DL) to classify the stages of damage and detect early stages can be a good alternative to current approaches.

In this article, a generative‐adversarial network (GAN) was developed to classify the stages of tree bark beetle infestation based on a small set of multispectral images.

## 2 RELATED WORKS

Both classical ML methods and deep learning methods are used to determine the phase of tree bark beetle infestation.

For example, Abdullah et al. (2019), Duračiová et al. (2020), and Trubin et al. (2022) use the linear regression (LR) method to determine the green stage of tree damage. Although this method is widely used in various industries and is easy to apply, linear regression assumes a linear relationship between data, which may be incorrect for this task. Linear regression models can be prone to overfitting if the model is too complex compared to the amount of data. They can also be sensitive to noise in the data and outliers, which can lead to inaccurate predictions.

Another popular method for assessing the stage of tree damage is the random forest (RF) method. In Foster et al. (2017), Honkavaara et al. (2020), Bárta et al. (2021), and Junttila et al. (2022) this method has been used to investigate trees damaged by bark beetles. The advantages of the random forest method include its speed, noise tolerance, and resistance to overfitting. However, successful application of this method requires in‐depth knowledge and experience in the domain under study. The random forest method is based on manual feature selection and requires the use of mathematical models to analyze the data. These models may have limited ability to generalize results to new or complex data.

Other popular machine learning methods used to detect trees affected by bark beetles include support vector machines (SVMs) (Abdollahnejad & Panagiotidis, 2020; Fassnacht et al., 2012, 2014), which are computationally complex and inefficient on noisy datasets; k‐nearest neighbors (KNNs) (Koreň et al., 2021; Näsi et al., 2015), whose performance is very sensitive to the dataset; maximum likelihood (Cessna et al., 2021; Klouček et al., 2019; Wulder et al., 2006), which requires prior knowledge of the structure of the data under study; and others.

As for deep learning methods, approaches to the task of determining the phase of tree bark beetle damage can be divided into categories: binary and multi‐class classification, semantic segmentation, and detection.

Thus, in Kapil et al. (2022), a convolutional neural network was developed to classify read green blue (RGB) images of tree crowns into 4 classes according to the phase of tree bark beetle damage.

In Minařík et al. (2021), Safonova et al. (2019), and Safonova et al. (2022), various approaches to detecting trees affected by bark beetles in images of certain forest areas and their classification using various convolutional neural networks such as YOLOv2, YOLOv3, YOLOv4, Custom‐convolutional neural network (CNNs), DenseNet‐1, etc. were presented.

In Chiang et al. (2020) and Kislov et al. (2021), segmentation methods were developed to identify affected forest areas.

The advantages of using neural networks are that they can work with data without complex feature preprocessing and without prior knowledge of the data structure, and convolutional networks can perform well and generalize data well, which can be very useful in the task of determining the degree of tree bark beetle damage. As for the disadvantages, CNNs usually require large training datasets in order to perform well. Therefore, training of deep convolutional neural networks is computationally intensive and takes a long time.

To overcome these disadvantages, this article proposes a generative‐adversarial network for classifying multispectral images of trees affected by bark beetles, which is trained on a small dataset.

Generative‐adversarial networks were first introduced in Goodfellow et al. (2014). A GAN is a neural network consisting of two networks: a generator (G) and a discriminator (D). The idea of such a network is that the generator is fed with random noise, from which it generates a certain image, and the discriminator is fed with an image and its task is to distinguish the image generated by the generator from the real image from the training dataset. The training of a GAN network takes the form of a competition between the Generator and the Discriminator in a zero‐sum minimal game for two which can be described by formula (1)

(1) $$\min_{G}\max_{D}V\left(D,G\right)=E_{x\sim p\left(x\right)}\left[\log D\left(x\right)\right]+E_{z\sim p\left(z\right)}\left[\log\left(1-D\left(G\left(z\right)\right)\right)\right]$$

where 𝑥~𝑝(𝑥) is an example of data from the real data space, and 𝑧~𝑝(𝑧) is a noise vector.

The training of both networks ends when they can no longer improve their current state. This happens when the generator produces data that cannot be distinguished from the data in (𝑥) and the discriminator no longer sees the difference between the generated and real data. This state is called the Nash equilibrium (Bang & Shim, 2018).

Since conventional GANs cannot be used in classification tasks, certain improvements have been developed for them. Thus, in Mirza and Osindero (2014), a Conditional GAN (CGAN) network was presented, in which the inputs of the generator and discriminator are fed not only random noise and images, respectively, but also class labels, which make the generator learn to generate images according to the labels passed to it. Also, in order to obtain a classifier, it is necessary that the discriminator can not only distinguish whether the image is real or generated but also to which class it belongs. For this purpose, AC‐GAN was proposed in Odena et al. (2017).

The idea for the development of this network was derived from Lin et al. (2018) and Kerdegari et al. (2019), where generative‐adversarial networks for classifying hyperspectral and multispectral images were presented. Here, a discriminator acts as a classifier. The main idea is that the generation of fake data partially compensates for the small size of the training data, which in turn improves the classification accuracy. Therefore, in this article:

- For the first time, a generative‐adversarial network was used to classify multispectral images of trees affected by bark beetle;
- The architecture of the generator using Channel Attention blocks is proposed, with which the discriminator achieves 87% classification results.

## 3 MATHEMATICAL STATEMENT OF THE PROBLEM

Given: X—a set of 64×64×5 multispectral images of trees, a set of class labels Y for images X, where y_i_ is the class label for the i‐th image of X, y_i_ ϵ {0,1,2}, where 0—healthy trees, 1—trees affected by bark beetle, and 2—dead trees.

The task is to classify the stages of tree damage by bark beetles based on multispectral image processing. This is a multi‐class classification problem, where each tree image belongs to one of three classes: healthy, bark beetle affected, and dead trees. Usually, in the problem of tree bark beetle damage, the data are divided into four classes, but in this work, we used a dataset in which the data are labeled into three classes corresponding to green, red, and gray stages of damage.

The task is to train a model f(*x*) (a classification function that takes an image x ϵ X and returns a predicted class label) that minimizes the classification error on the dataset X.

Although convolutional neural networks are often used for such tasks, their application in this area is limited due to the need for large amounts of training data. Collecting such data for the problem of determining the degree of tree bark beetle damage is difficult, expensive, and time‐consuming.

To solve this problem, it was decided to use a conditional generative‐adversarial network (CGAN), but when training CGAN on a small dataset, the problem of mode collapse arises for classes of generated images.

To solve the problems described above, we use GAN to classify the stages of tree bark beetle damage. The GAN consists of two neural networks: a generator G and a discriminator D.

The generator G takes a random noise vector z and a label class value and generates an image of the given label class. The idea is that the still small size of the training dataset is compensated by the labeled data generated by the generator.

The discriminator D accepts an image x and returns the probability that x belongs to the training dataset X and the probability of the class to which the image belongs.

In this network, the role of the classifier is played by a discriminator trained on training data and data generated by a generator that has learned to generate data from the distribution of training data.

To solve the mode collapse problem, we use stepwise learning with a transition from unconditional to conditional learning as proposed in Shahbazi et al. (2022).

## 4 DATA DESCRIPTION

Multispectral images of trees affected by the bark beetle at different stages of damage are used to train the neural network (Junttila et al., 2024). The data were provided by Samuli Junttila, Associate Professor at the University of Eastern Finland School of Forest Sciences, who is a co‐author of this article. The data provided included multispectral and RGB orthomosaics of the territories and a dataframe with the characteristics and locations of the marked trees in the corresponding images.

**FIGURE 1:**
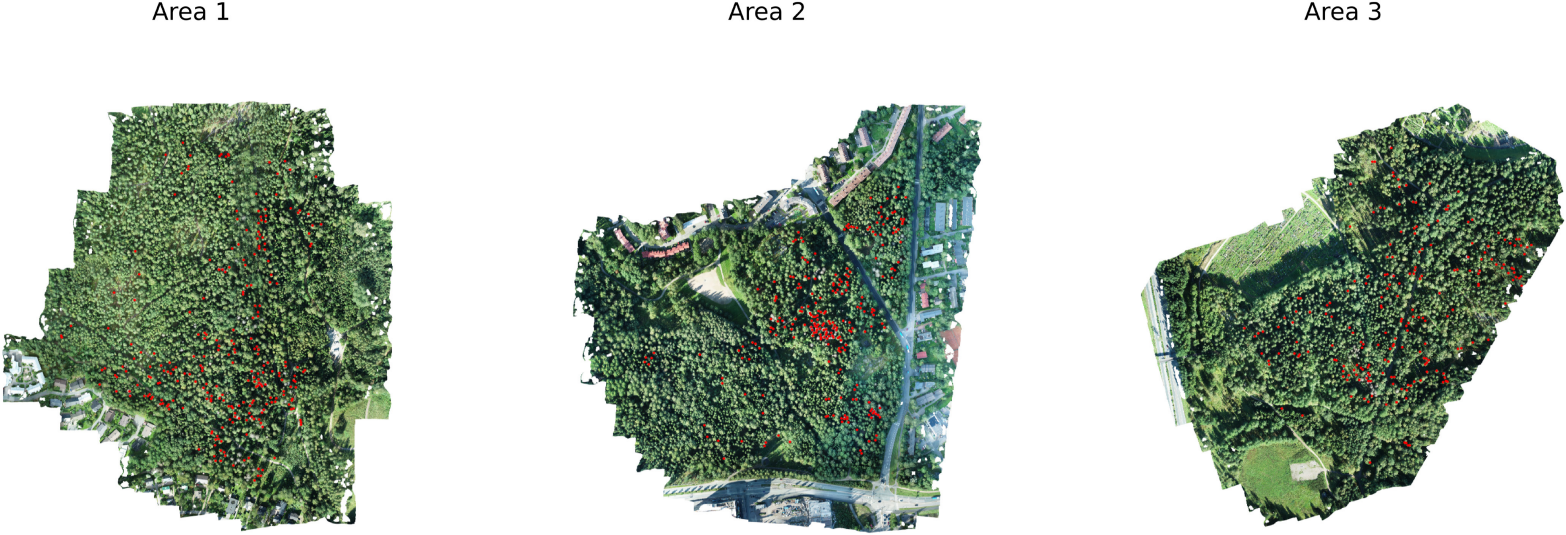
RGB images of territory.

**FIGURE 2:**
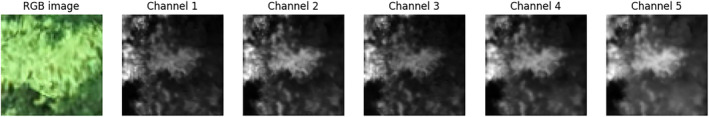
RGB and multispectral images of a healthy tree.

**FIGURE 3:**
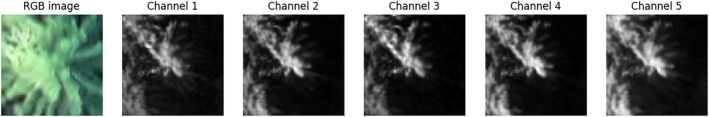
RGB and multispectral images of an affected tree.

**FIGURE 4:**
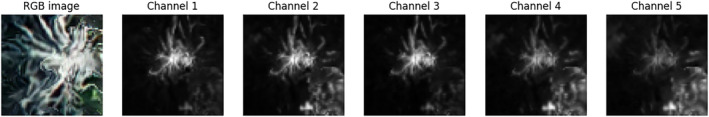
RGB and multispectral images of a dead tree.

To obtain a set of multispectral images of trees, 3 multispectral orthomosaics of the territory were used (Figure 1) (in this figure, red dots indicate labeled trees from the provided dataframe):

These images were collected using a Phantom 4 Pro V2.0 drone with a Micasense RedEdge M multispectral camera. The ground sampling step size (GSD) was approximately 8 cm for RedEdge. The RedEdge has five cameras that capture spectral bands: blue (center wavelength: 475 nm; bandwidth: 32 nm), green (560; 27 nm), red (668; 14 nm), edge red (717; 12 nm), and near infrared (NIR) (842; 57 nm) (Junttila et al., 2022). Images of the trees specified in the dataframe were cut from these images. The size of the resulting multispectral images was 64×64×5, where 5 is the number of channels. Examples of RGB and multispectral images of trees at different stages of bark beetle damage are shown in Figures 2, 3, 4:

The stage of tree damage by bark beetle was assessed based on the analysis of the crown and trunk. The crown was graded by leaf color into six different classes (scores 0–5): healthy green, slightly faded green, yellowish, yellow, red‐brown, and gray. Defoliation was also assessed in five classes (scores 0–4): 0%–10%, 10%–25%, 25%–50%, 50%–75%, and 75%–100% and crown size. Significantly smaller vertical crown size was considered an indicator of lesion (scores 0–1). Scores were assigned depending on the condition, with zero given to the healthiest class and one point for each class in the direction of more severe symptoms. The trunks were also evaluated for the presence of fresh resin flows (indicating bark beetle damage) and bark damage at the structural level into three classes (scores 0–2): not detected, moderate damage, and severe damage.

Tree health classification was based on symptoms assessed in the field by multiplying the scores for crown discoloration and shedding by 1.5 and summing all scores for each tree. Crown symptoms were weighted because they are easier to detect from images taken from above. Every tree that had a score of two or less was classified as healthy, and every tree with more than two points was classified as a declining tree. In addition to healthy and declining trees, visually dead trees were identified and classified using RGB images of the trees that were collected for the study. Trees with reddish, brownish, or gray crowns were considered dead. (All symptom scores of the respective trees were provided in the dataframe.).

After creating, classifying, and balancing the multispectral image set, a set of 342 images (114 images of each class) was obtained from this data, and the network was trained.

## 5 MULTISPECTRAL IMAGE PROCESSING

Since both the generator and the discriminator of our GAN network are conventional convolutional networks that generate/classify three‐channel images, it was necessary to select 3 channels out of 5 that are most important for classification.

In the work of Junttila et al. (2022) normalized difference indexes (NDI) were used between wavelengths in the layers calculated using formula (2) to process multispectral images:

(2) $${\mathrm{NDI}}_{1,2}=\left(\rho_{1}-\rho_{2}\right)/\left(\rho_{1}+\rho_{2}\right)$$

where ρ is the wavelength of a particular layer. In Junttila et al. (2022), it was also noted that the most informative features were the following: ${\mathrm{NDI}}_{\mathrm{red}-\text{edge},\mathrm{red}}$, ${\mathrm{NDI}}_{\mathrm{red},\mathrm{NIR}}$, and ${\mathrm{NDI}}_{\mathrm{red},\text{green}}$ therefore, to train the neural network, pixel‐by‐pixel NDI values for the corresponding layers were calculated and thus an image of size 64×64×3 was obtained, where the 3 layers were respectively ${\mathrm{NDI}}_{\mathrm{red}-\text{edge},\mathrm{red}}$, ${\mathrm{NDI}}_{\mathrm{red},\mathrm{NIR}}$, and ${\mathrm{NDI}}_{\mathrm{red},\text{green}}$.

## 6 THE PROPOSED METHOD

**FIGURE 5:**
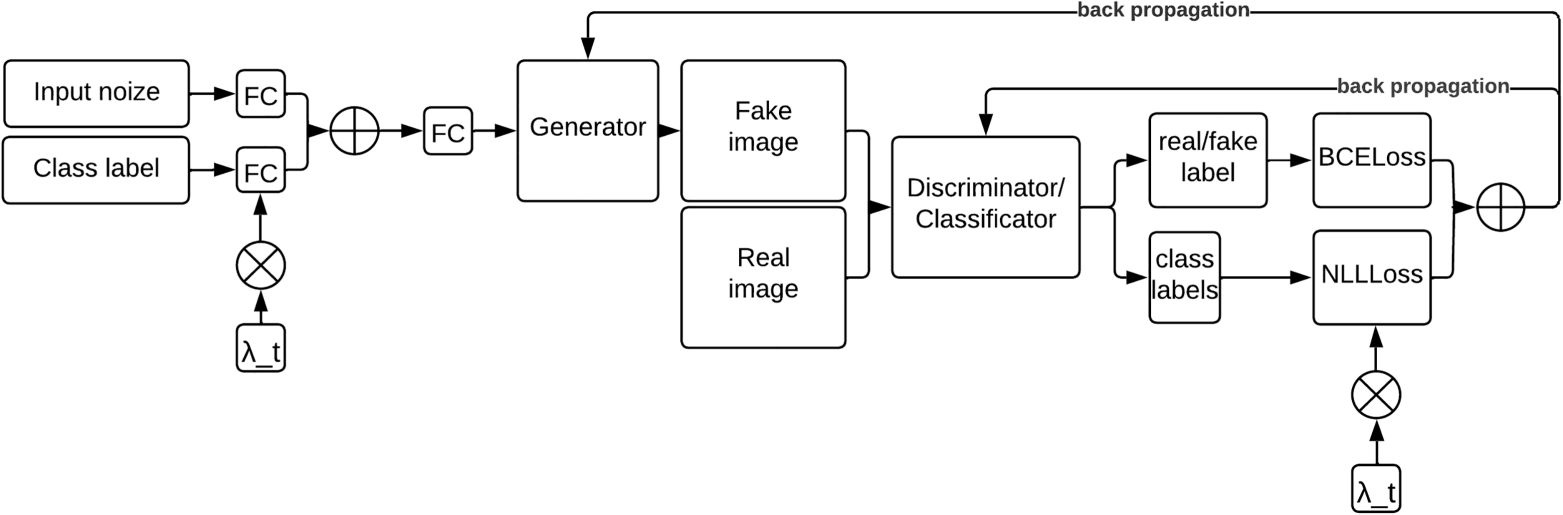
General architecture of a generative adversarial neural network.

Convolutional neural networks are commonly used to solve the problem of image classification, but training conventional convolutional neural networks requires large sets of labeled data, which is a rather expensive and time‐consuming task. Therefore, instead of a conventional convolutional network, we developed a generative‐adversarial network in which the problem of a small number of images in the training dataset is solved by the fact that the trained generator will generate images of the specified classes that cannot be distinguished from the real ones, which will create additional data for training the classifier (Figure 5).

To solve the problem of multispectral image (MSI) classification, a GAN with the following architecture was chosen:

We based our approach on the GAN model introduced by Lin et al. (2018). However, the problem with this network was that it was designed to train on a large dataset, and when applied to a dataset with a small number of images, the network began to suffer from the mode collapse problem. Mode collapse is a problem that can arise when training a GAN when the generator generates only a limited number of different images (e.g., only one specific image for each class). This is a problem because the main task of the generator is to generate a variety of images on which the classifier can learn.

To overcome the problem of mode collapse in CGANs with a small amount of training data, we applied the idea presented in Shahbazi et al. (2022). To achieve this, the network training was divided into three stages. The first stage, unconditional learning, involves the generator learning to produce images without considering their class labels, while the discriminator focuses solely on distinguishing real images from generated ones. During the second transition stage, the weight assigned to class label values in the loss functions gradually increases. Finally, in the third stage, conditional learning, the weight reaches a value of 1, allowing the generator to produce images conditioned on specific class labels.

**FIGURE 6:**
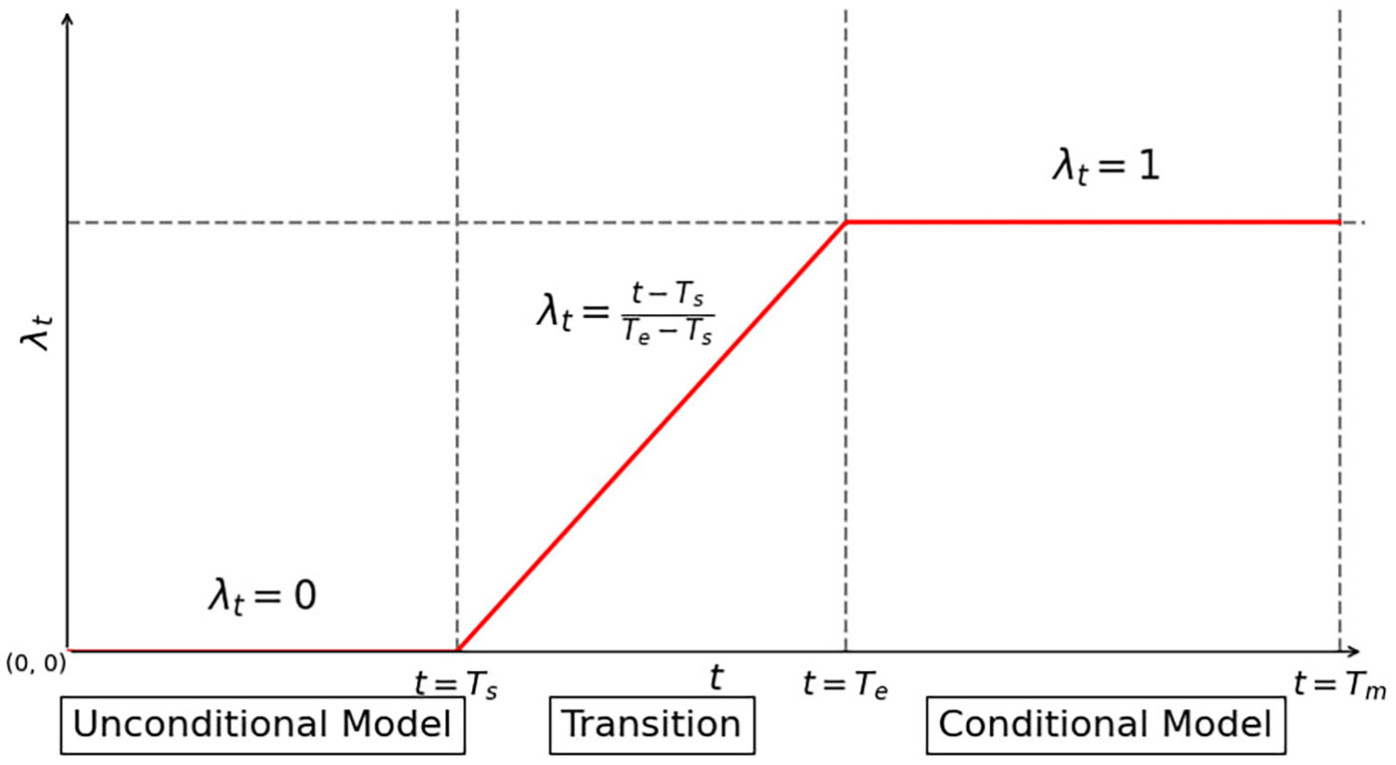
Visualization of the transition function *λ*_t_. *T*_s_, *T*_e_, and *T*_m_ denote the moment (epoch) of the beginning of the transition, the end of the transition, and the number of epochs in training, respectively. The visualization is reproduced from the Shahbazi et al. (2022).

To implement the transition process, the transition function λ_t_ is introduced (visually represented in Figure 6):

(3) $$\lambda_{t}=\min\left(\max\left(\frac{t-T_{s}}{T_{e}-T_{s}},0\right),1\right)$$

where $T_{s}$ is the epoch number where the transition starts, and $T_{e}$ is the number of the epoch where the transition ends.

The value of λ_t_ acts as a weight for the class labels for the generator input data, and for the conditional part of the generator and discriminator input function:

(4) $$L^{G}=L_{\mathrm{UC}}^{G}+\lambda_{t}*L_{C}^{G}$$

(5) $$L^{G}=L_{\mathrm{UC}}^{D}+\lambda_{t}*L_{C}^{D}$$

here $L_{\mathrm{UC}}^{G}$ and $L_{\mathrm{UC}}^{D}$ are the unconditional parts of the loss functions of the generator and discriminator, and $L_{C}^{G}$ and $L_{C}^{D}$ are the conditional parts of the generator and discriminator loss functions.

When the epoch number $t<T_{s}$ is equal to $\lambda_{t}=0$, the network is trained in unconditional mode. When $T_{s}<t<T_{e}$ $-0<\lambda_{t}<1$, the transition occurs. When $t>T_{e}$ $-\lambda_{t}=1$, the network switches to the conditional learning mode.

## 7 ARCHITECTURE OF THE GENERATOR AND DISCRIMINATOR

### 7.1 Generator

This network is a generator in a neural network used to generate images. Different network architectures can be used to generate images. In this work, a deep neural network with backpropagation layers, which can be used to increase the size of images, and Channel Attention blocks, which allow the generator to highlight more important channels in feature maps, were used as a generator. This architecture was chosen because of its relative simplicity of implementation and the high quality of the generated images.

Let's look at a detailed description of the topology of this network.

1. Input layer

- The size of the input noise vector (usually from a Gaussian distribution) is “nz.”
- The image class label is also entered as a vector, for example, a one‐hot encoding with the size “nb_label.”
- These two parts of the input data are concatenated to create a common vector “x” containing both noise and information about the class of objects to be generated.

2 The first block

- After concatenation, the input tensor passes through the inverse convolution layer (ConvTranspose2d), which transforms the “nz + nb_label” channels into an output with the size “ngf * 8.”
- After that, the Batch Normalization layer is applied and the ReLU activation function for nonlinearity is applied.

3 Further blocks

- The next three blocks also consist of inverse convolution layers (ConvTranspose2d), each of which reduces the number of channels and doubles the image size.
- After each inverse convolution, the Batch Normalization layer is applied and the ReLU activation function is applied for nonlinearity.
- After the second and third blocks are the Channel Attention blocks.

4 The output unit

- The last block has a reverse convolution that converts the “ngf * 2” channels to an output with a size of “nc” (usually “3” for RGB).
- After the reverse convolution, the Tanh activation function is applied to ensure that the pixel values are in the range of −1 to 1, which is suitable for images.

### 7.2 Channel attention

Channel Attention is a mechanism in neural networks that helps the model learn to focus on the most important channels in the input data (or intermediate representations). Channel Attention allows the network to dynamically weight these channels, emphasizing those that are more relevant to generating the desired images.

**FIGURE 7:**
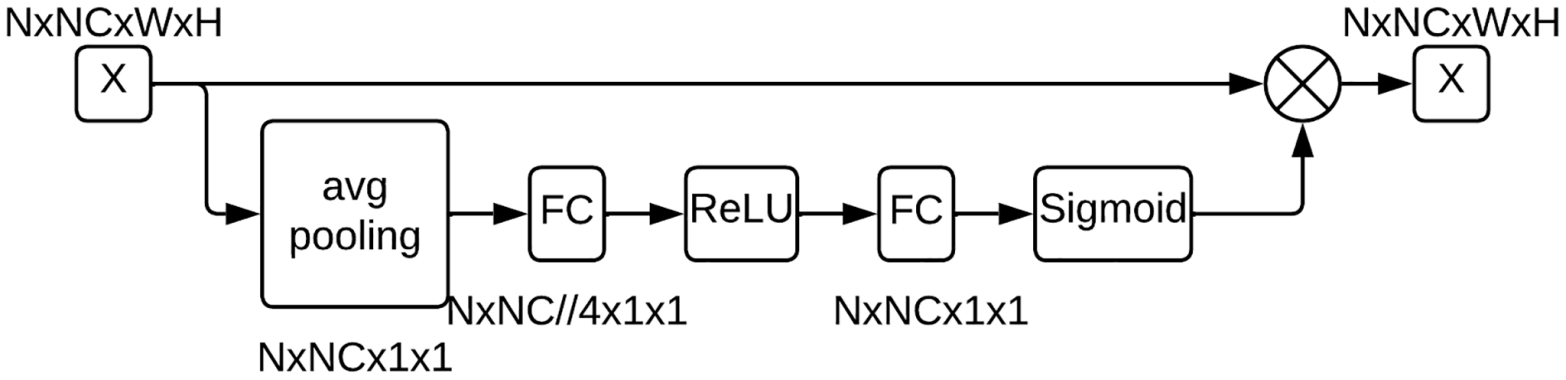
Architecture of the Channel Attention block. Here, “avg pooling” is average pooling layer, “FC” is fully connected layer, “ReLU” and “Sigmoid” are activation functions, and “⊗” is an elementwise multiplication. NxCxWxH represents the shape of the input data and corresponds to the batch size, number of channels, width, and height, respectively.

Architecture of the Channel Attention block (Figure 7):

- Reducing the spatial dimensions of each input channel to a single value while preserving the average information on that channel.
- Fully connected convolutional layer reduces the number of channels by reducing the dimensionality using a reduction factor.
- Application of the nonlinear activation function ReLU.
- Fully connected convolutional layer restores the original number of channels.
- Apply a nonlinear Sigmoid function that gives weights between 0 and 1 for each channel.
- The resulting attention weights are multiplied by the corresponding channels of the input feature map, increasing important channels and suppressing less important ones.

**FIGURE 8:**
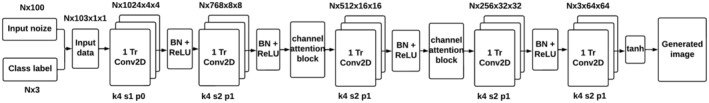
Generator topology. Here, “1 Tr Conv2D” refers to a transpose convolution layer, “BN” stands for batch normalization layer, and “ReLU” and “tanh” are activation functions. NxCxWxH represents the shape of the input data, corresponding to the batch size, number of channels, width, and height, respectively. *k*, *s*, and *p* are hyperparameters of the transpose convolution layer and represent the kernel size, stride, and padding, respectively.

In general, the generator architecture generates images by combining noise and class information, passing them through several layers of inverse convolution with decreasing number of channels and increasing image size, and through Channel Attention blocks. At each step, Batch Normalization and ReLU layers are applied to improve the robustness and nonlinearity of the model (Figure 8).

At this stage, the following parameter values were used: nz = 100, ngf = 128, nc = 3, nb_label = 3.

### 7.3 Discriminator

This network acts as a discriminator in a neural system used to evaluate and recognize generated images. The choice of a deep convolutional neural network is due to their high efficiency in classification tasks.

Let's look at a detailed description of the topology of this network.

1. Input layer

- The image is entered as a tensor with “nc” channels (usually “3” for RGB).

2 The first block

- The input tensor is passed through a conventional convolution (Conv2d), which transforms “nc” channels into an output with size “ndf.”
- After that, the LeakyReLU activation function is applied to introduce nonlinearity.

3 Further blocks

- The next four blocks consist of conventional convolution (Conv2d), Batch Normalization layers, LeakyReLU activation function, and Dropout for regularization (after the second convolution layer).
- The number of channels increases with each block, which helps the network detect more and more complex features in images.

4 The output unit

- The last block has a conventional convolution (Conv2d), which converts “ndf * 8” channels into an output with the size of “ndf * 2.”
- After the reverse convolution, the LeakyReLU activation function is applied.

5 Evaluation

- After convolution, the output tensor is converted to a vector using “view,” which becomes the input for two linear layers: “disc_linear” and “aux_linear.”
- “Disc_linear” gives one value for the authenticity assessment (0‐fake, 1‐real) and applies the Sigmoid activation function.
- “Aux_linear” produces a probability vector for each class of labels (the softmax activation function is used).

**FIGURE 9:**
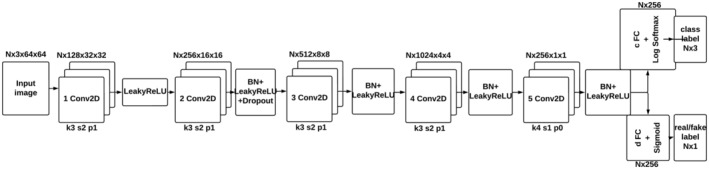
Discriminator/Classifier topology.

Hence, this discriminator architecture has several conventional convolutional layers with different numbers of channels and activation functions to detect and classify image features (Figure 9).

At this stage, the following parameter values were used: nz = 100, ndf = 128, nc = 3, nb_label = 3.

## 8 LEARNING PROCESS AND HYPERPARAMETERS

The network training process consists of several stages:

### 8.1 Initialization of hyperparameters and optimizers

Hyperparameters:

*Learning rate*:

lr_D = 0.001.

lr_G = 0.01.

*Weight decay*:

wd = 0.0005.

decreasing_lr = “60,120,240,620,800,1200.”

pretrain epochs = 50.

*T*_s_ = 500.

*T*_e_ = 600.

Optimizers: for the discriminator (optimizerD) and the generator (optimizerG). Here we use Adam optimizer with the specified hyperparameters lr, wd.

### 8.2 Consistent training

For each training iteration:

1. Zeroing the gradients of optimizers.
2. Transferring real images to the discriminator D to assess their authenticity and classify them. The returned values (s_output, c_output) are used to calculate the loss of the discriminator.
3. Generating images by generator G and passing them to the discriminator for evaluation. The returned value (fake) is used to calculate the loss of the discriminator.
4. Calculating the loss for the discriminator (s_criterion + c_criterion) based on the discriminator's predictions for real and generated images.
5. Backpropagation and updating the discriminator parameters using the optimizerD.
6. Generate new images with the generator.
7. Sending the generated images to the discriminator and classifier for classification.
8. Backpropagation and updating of the oscillator parameters using the optimizer (optimizerG).

This process is repeated for several epochs (training iterations) until the classifier's accuracy is satisfactory.

### 8.3 Loss functions

The above code uses two different loss functions: s_criterion for the discriminator and c_criterion for the classifier.

Let's describe each of these loss functions:

#### 8.3.1 Loss function for the discriminator (s_criterion)

The binary cross‐entropy function (BCELoss) is used, since the task of the discriminator is to classify images into real and generated ones.

This loss function calculates the loss between the predicted probabilities of the discriminator and the target values (1 for real images and 0 for generated images).

The value returned by this loss function reflects how far the discriminator's predictions differ from the correct labels.

#### 8.3.2 Loss function for the classifier (c_criterion)

The negative log‐likelihood loss function (NLLLoss) is used, since the classifier's task is to classify images into different classes.

The NLLLoss function calculates the loss between the predicted probabilities and the target labels. It takes as input the logarithms of the probabilities (or, respectively, the predicted logits) and the target class labels.

The value returned by this loss function reflects how far the classifier's predictions differ from the correct labels.

Both of these functions are used to calculate losses in the model training process, and their purpose is to ensure that the model is trained to make accurate predictions for real and generated images.

## 9 RESULTS

To train the network, the pre‐processed images were divided into training and test sets in an 8:2 ratio. The training was performed for 1250 epochs.

As a result of training the network described above, the following metric values were obtained, as shown in Table 1.

**TABLE 1:** Training results.

Metric	Value (%)
Accuracy	87.5
Recall	87.1
Precision	87.2
Kappa	73.3
F1	86.7
F2	87.1

Additionally, for comparison, classifiers were trained separately without using generator model on the original training data without augmentation (WA) and on the augmented data with augmentation (A). The results were compared to those of the Random Forest method used in Junttila et al. (2022), as shown in Table 2.

**TABLE 2:** Comparison of results.

Method	Accuracy (%)
Random Forest	84.5
Classifier (WA)	75
Classifier (A)	85.1
**GAN model**	**87.5**

## 10 DISCUSSION

The results of this study highlight the significant potential of generative‐adversarial networks (GANs) for addressing the challenges of detecting and classifying tree bark beetle damage using multispectral imagery. With an overall classification accuracy of 87.5%, the GAN‐based approach outperforms traditional machine learning techniques such as Random Forest (84.5%) and standard classifiers trained with data augmentation (85.1%). This performance is especially noteworthy given the limited dataset, underscoring the strength of GANs in generating additional synthetic training data to improve model robustness.

One of the critical advantages of this method lies in its ability to reduce dependence on large, labeled datasets, which are often difficult and expensive to acquire, particularly for forestry applications. The integration of Channel Attention blocks further enhances the model by allowing it to focus on the most informative spectral bands, thereby improving the accuracy of damage classification. This combination of data generation and selective focus on spectral features enables the system to detect subtle changes in tree health, particularly during the early stages of bark beetle infestation, which is crucial for timely interventions.

However, despite these promising results, the study also reveals certain limitations. First, the model was tested on a relatively small and homogeneous dataset, which may not fully capture the variability present in larger or more diverse forest ecosystems. Therefore, future work should focus on validating the system across broader datasets encompassing different tree species, geographic regions, and environmental conditions. Additionally, while the generated synthetic data improves performance, there is always a risk that these data do not fully represent the complexities of real‐world scenarios. Further research is necessary to ensure the generalizability of these synthetic data across different contexts.

Another limitation of this study is the computational demand of GANs. Training GANs, particularly when combined with CNNs, can be resource‐intensive, requiring significant computational power and time. While this may be less of a concern in research settings, it could pose challenges for real‐time applications in forest management, where rapid deployment and low‐cost solutions are often prioritized.

In conclusion, while there are challenges to overcome, the development and refinement of GAN‐based models for forest health monitoring offer significant promise. With further improvements and validation, these tools could play a pivotal role in early detection systems for forest management, helping mitigate the economic and ecological impacts of bark beetle infestations.

## 11 CONCLUSION

In conclusion, this research demonstrates the successful application of generative‐adversarial neural networks to the task of detecting and classifying bark beetle damage using multispectral images. The developed system provides a significant improvement over traditional methods, achieving a high classification accuracy of 87.5%. By generating synthetic training data, the GAN approach addresses the challenge of limited datasets, and its integration with Channel Attention mechanisms enhances its focus on critical spectral features.

While promising, further research is needed to validate the system across more extensive datasets and forest ecosystems. Future work could also explore the integration of this system into real‐time forest monitoring tools, offering forestry managers a powerful means to detect beetle infestations early and take preventative action. This could result in substantial economic benefits for the forestry industry and contribute to the conservation of vital forest ecosystems.

## CONFLICT OF INTEREST STATEMENT

The authors declare no conflict of interest.

## Data Availability

The data that support the findings of this study are openly available in "Data for estimating spruce tree health using drone‐based RGB and multispectral imagery" at https://zenodo.org/records/13925862, reference number 16.
